# From visit counts to quality care: A qualitative study on early implementation of a new model for antenatal care in Kavre district, Nepal

**DOI:** 10.1371/journal.pgph.0005076

**Published:** 2026-10-05

**Authors:** Samita K C, Rajani Shakya, Abha Shrestha, Abha Shrestha, Emma Radovich

**Affiliations:** 1 Research and Development Division, Dhulikhel Hospital, Kathmandu University Hospital, Dhulikhel, Nepal; 2 Department of Community Medicine, Kathmandu University School of Medical Sciences, Dhulikhel, Nepal; 3 Department of Gynecology and Obstetrics, Dhulikhel Hospital, Kathmandu University Hospital, Dhulikhel, Nepal; 4 Faculty of Epidemiology and Population Health, London School of Hygiene and Tropical Medicine, London, United Kingdom; Washington University in St. Louis, UNITED STATES OF AMERICA

## Abstract

In 2016, WHO updated its antenatal care (ANC) guidelines, increasing the minimum number of ANC visits from four to eight and recommending practices for those visits. Nepal adopted this new ANC model in 2022, but limited research has examined how the model has been implemented. This study explored the perspectives of pregnant women, ANC providers and local policymakers on the rollout of the new ANC model in Kavre district of Nepal. We conducted an exploratory qualitative study involving interviews with pregnant women, ANC providers and local policymakers along with semi-structured facility observations. Six primary care level government health facilities offering ANC services were purposively selected from three municipalities of Kavre district, Nepal. Data included eight interviews with pregnant women, seven with providers and three with local policymakers along with field notes from observations in six facilities. Interview transcripts and observation field notes were coded manually and analyzed thematically. Three major themes emerged from the analysis of the interviews and observations. Providers and policymakers viewed the new ANC model mainly as increase in visit frequency for documentation rather than enhanced quality care. Although orientation on the increased number of visits was presented to providers, most providers lacked understanding on the content and purpose of each visit. Pregnant women, particularly those in rural areas, reported logistical, financial and physiological challenges in completing all recommended visits and accessing care at referral sites. Implementation of new ANC model in Kavre district largely remained focused on visit quantity rather than quality. Strengthening provider’s capacity, ensuring availability of guidelines, enhancing understanding of its purpose and implementation, and fostering person centered care are essential to achieve the intended benefits of the model.

## Introduction

Antenatal care (ANC) is a crucial aspect of maternal healthcare, providing essential care and support to women during pregnancy. ANC offers a platform for timely interventions to prevent perinatal complications by providing health education, disease prevention, early detection and treatment, and birth preparedness.

Until 2016, the World Health Organization (WHO) had recommended a Focused Antenatal Care (FANC) model that included recommended interventions delivered over at least four ANC visits during the pregnancy. This recommendation was based on evidence at the time suggesting that similar maternal and fetal outcomes were achieved with a minimum of four targeted ANC visits compared to models with higher numbers of visits [1]. The WHO updated its ANC guidelines in 2016 to recommend care and interventions to promote a positive experience and outcome of pregnancy and increased the minimum number of visits from four to eight during pregnancy [2]. This new ANC model highlighted that women’s interactions with ANC providers should be opportunities for comprehensive care which would include medical interventions and monitoring, with an equal emphasis on timely information and psychosocial and emotional support by care providers [3]. The changes were informed by evidence that more frequent ANC visits, particularly at later stages of pregnancy, can improve the detection and management of complications such as hypertensive disorders which remain a leading cause of maternal mortality [2,4].

Nepal adopted the new ANC model in 2022 and recommended the interventions outlined in the WHO guideline as a minimum, acknowledging that these increased ANC visits from four to eight, would result in timely detection of health problems [5]. While the recommended timing of ANC visits is provided, the WHO guidelines and the Nepal National Medical Standard for Maternal and Newborn care guidelines did not define the content of each of those visits. A 2022 facilitation document, the “ANC to PNC continuum of care guideline” was developed by Family Welfare Division of Ministry of Health and Population in Nepal and provided detailed information regarding timing of visits and contents on history taking, examinations, interventions, health promotion activities and actions to be carried out on each visit [6].

Nepal has made significant progress in ANC attendance with the help of various health policies and programs, but quality of care remains a concern [7]. Coverage of at least 4 ANC visits increased significantly from 53% in 2011 to 81% in 2022 [8]. Despite the high coverage of 4 + ANC visits documented in the 2022 Nepal Demographic Health Survey (NDHS), few women (6%) reported having more than eight ANC visits during their most recent pregnancy in the two years before the survey, which was conducted just prior to the rollout of new ANC model [5,8]. The Nepal Safe Motherhood and Newborn Health Road Map 2030 described key issues hindering the quality of ANC, including poor service readiness, lengthy waiting times, and inadequate communication from providers and challenges linked with the provider’s workload, availability of training and skills [7].

There is limited evidence on how the WHO recommended new ANC model is being implemented in health facilities in Nepal. Other studies in low-or middle-income countries have examined implementation of the new ANC model, finding substantial gaps in content and challenges in delivery [9,10]. Previous research on the new ANC model has focused on acceptability among pregnant women, prevalence and adherence to more than eight ANC visits [11–15]. Yet, programs to improve maternal health are both technical and social interventions [16]. Understanding the implementation of the new ANC model requires attention to the social organization of the work from multiple stakeholders, which Normalization Process Theory describes as “the things that people do to perform certain acts and meet specific goals” [17]. To our knowledge, this is the first study in Nepal to explore implementation of the new ANC model, incorporating views from policymakers, providers and pregnant women.

This study aimed to provide insights into the rollout of the new ANC model from the perspective of pregnant women, ANC providers and local policymakers in Nepal. This will aid in identifying implementation gaps between policy and the experience on the ground.

## Materials and methods

### Ethics statement

Written informed consent was taken before the interviews from each participant, and written informed consent with facility in charge was taken prior to the facility observations. Ethical approval for the study was obtained from the Nepal Health Research Council (Reg No: 635/2023), Kathmandu University Institutional Review Committee (Approval No: 85/24) and the London School of Hygiene and Tropical Medicine (Ethics Ref: 29823). The voluntary participation of all the study participants was ensured throughout the study process. To ensure privacy and confidentiality, all interviews and observation field notes were assigned anonymized personal and facility identifiers. Data were stored on a secure password-protected laptop and access was restricted to the core research team.

### Research context and study design

This exploratory qualitative study was conducted as a sub-study of a mixed-method research project examining hygiene preparedness and urine testing practices in pregnancy in health facilities offering ANC services. The study presented here emerged inductively during early data collection for the larger project and was conducted using an exploratory qualitative design involving face-to-face semi-structured interviews with pregnant women, ANC providers and local policymakers. This study also drew from qualitative field notes from a semi-structured facility observation checklist administered at the participating health facilities.

ANC services in Nepal are provided in all public health facilities including community-level primary healthcare outreach clinics [18]. The larger project, of which this study was part, was conducted in three municipalities (two urban and one rural) of Kavre district, Bagmati province. At the point that this study was conducted in 2024, all municipalities had started implementing the new ANC model.

### Site selection and facility description

We purposively selected six primary level government health facilities from the three municipalities. Selection of the facilities was done in consultation with municipal health officials and with the aim of conducting research for the larger project (of which this study is part) in facilities that were considered important for ANC provision in the local area and that offered variation in service availability. Facilities with higher ANC caseloads were selected, and the selection of health facilities aimed to capture variation in geographic characteristics and the presence of a birthing unit and laboratory. Two health facilities were selected in each municipality (a total of six facilities). The characteristics of selected health facilities are presented in Table 1.

**Table 1:** Characteristics of study facilities.

Facility ID	Facility type	Location	Total Population served	No. of ANC Providers	Birthing Unit	Laboratory
F1	Primary Health Care Center	Urban	4535	3	Yes	Yes
F2	Health Post	Urban	7786	1	No	No
F3	Municipal Hospital	Urban	4408	3	Yes	Yes
F4	Health Post	Rural	3035	2	Yes	Yes
F5	Health Post	Rural	2935	2	Yes	Yes
F6	Health Post	Urban	4025	2	Yes	Yes

### Participant sampling and recruitment

Pregnant women, ANC providers and local policymakers were selected purposively for interview. Data collection was conducted by two members of research team, SK and RS, who directly approached the eligible study participants to explain the study purpose and obtain voluntary written informed consent. Interviews were conducted in private spaces within the health facility or government offices to ensure confidentiality. No financial incentives were provided to study participants, but refreshments were offered to acknowledge their time. The details about the study participant are as below:

#### Pregnant women.

Pregnant women visiting the selected health facilities who had attended for ANC in the health facility were approached to participate. Participants consenting to take part were at various stages of pregnancy and varied in parity. A total of eight pregnant women were interviewed.

#### ANC Providers.

Clinical staff who were directly involved in providing antenatal care at the selected facilities were eligible and approached to participate. In facilities where there was more than one ANC provider on site during data collection, the staff member whose role was primarily focused on ANC service delivery was approached to take part. A total of seven ANC providers were interviewed in the study.

#### Local policymakers.

Municipal level health officials who were responsible for maternal and child health programs, including implementation and monitoring of ANC, were eligible and approached for interview. A total of three local policymakers consented and took part in interviews to provide insights into ANC implementation.

Sample size was guided by principles of purposive sampling in identifying information-rich cases, and of data saturation within data collection resource constraints. Recruitment and data collection continued until no substantial new insights emerged from successive interviews (primarily relevant for the interviews of pregnant women and ANC providers), with later interviews primarily reinforcing existing themes suggesting that inductive thematic saturation was being reached [19]. The three local policymakers represented the key actors responsible for implementation and monitoring of ANC within the study area; they were included to explore relevant local policy perspectives rather than to seek thematic saturation amongst policymaker’s accounts.

### Data collection

Interviews and observations were conducted by two female researchers: SK- a sociologist and RS- a clinically trained researcher. We conducted semi-structured observations and face-to-face interviews in each participating health facility. We developed a semi-structured observation checklist to gain an in-depth understanding of the interaction between pregnant women and providers during ANC visits, and to collect information on facility physical infrastructure, including water and sanitation infrastructure (see S2 Text). Formal written consent to observe the health facility was obtained from facility managers. Researchers sought informal verbal consent from facility staff and patients encountered during the observations, explaining the purpose of the study. Researchers were sensitive to verbal and non-verbal expressions of discomfort and would pause observations when needed to protect patient privacy, including ensuring visual privacy during physical examinations. Observations were conducted for 2–3 working days in each of the six health facilities, prioritizing designated ANC days for observation and to aid in interview recruitment. Researchers visited different areas of the facility to assess infrastructure and would sit and observe staff and patient interactions in the waiting areas and ANC examination rooms. This approach of observation led to capturing real-time interactions and ANC environments to understand the contextual factors shaping ANC provision within selected health facilities. The observation checklists were completed prior to the interviews with providers.

All interviews were conducted in Nepali language, lasted approximately 30–60 minutes, and were based on topic guides prepared for each type of study participant (see S1 Text). The topic guides for pregnant women and ANC providers were pretested in the ANC clinic of a tertiary-level hospital in the district and adapted accordingly. The researchers also iteratively adapted the interview topic guides based on field notes collected during the observations. Reflection meetings with the research team were conducted after completing data collection in each facility, identifying further areas for investigation and probing in subsequent interviews.

The data collection was conducted from January to June 2024.

### Data analysis

All the interviews were audio recorded and transcribed verbatim. The transcriptions were conducted by native Nepali speakers. The interviews were analyzed in Nepali language to preserve linguistic nuances that could be lost in the translation. Initial codes were developed inductively by SK and were reviewed and discussed with the research team. A subset of the interviews (five interviews) was translated into English by an external translator and were quality checked by research team. Two translated transcripts were then double coded independently by SK and ER to enhance analytical robustness. Discrepancies in coding and interpretation were resolved through discussion during weekly team meetings, reflecting on different researchers’ perspectives and interpretation until consensus grounded within the data was reached. The analysis followed Braun and Clarke’s reflexive thematic analysis approach involving data familiarization, iterative coding, development and refinement of codes and themes [20]. Coding was done manually in MS Word, and data organization was managed using MS Excel. Relevant sections of the transcripts were translated into English for the write up of findings. Minor grammatical edits were made to improve readability without altering meaning of those selected sections of transcripts. The translated sections of the transcripts were quality checked and verified for meaning accuracy by the research team.

#### Theoretical framing.

This exploratory qualitative sub-study arose out of initial interviews with ANC providers and pregnant women identifying issues in new ANC model implementation, which was subsequently integrated into data collection. The research team initially explored the Theory of Acceptability (TFA) as a potential lens for interpreting stakeholders’ perceptions of the new ANC model [21]. However, as this work emerged within a broader study examining hygiene preparedness and urine testing practices in ANC settings, implementation frameworks such as the TFA were not considered during the development of data collection tools. During analysis, we explored the relevance of the TFA but found limited alignment between its constructs and the nature of our data. We therefore adopted an inductive thematic analysis approach, allowing themes to emerge directly from participants’ narratives rather than being constrained by a predefined framework [20]. While a theory-driven approach can enhance analytical consistency and comparability, inductive analysis enabled a more grounded interpretation that captured context-specific experiences and suited the exploratory nature of the study [22].

#### Triangulation.

Interviews of different types of participants were coded separately and responses of three different categories of respondents were arranged in data matrix to develop, compare and refine the themes. These themes were later also interrogated with observation field notes to triangulate findings.

A stakeholder meeting of policymakers including representatives from Nepal’s National Health Training Center, provincial health office, municipal health coordinators, and representatives from selected health facilities, ANC providers and researchers was conducted in July 2024 to share preliminary findings from the analysis. The insights from stakeholders were used to reflect on and validate initial interpretations and to refine the final version of results and recommendations.

### Researcher reflexivity

The majority of the interviews were conducted by the first author (SK), who had prior experience conducting interviews with women and key informants. The researcher acknowledged the potential influence of power dynamics between interviewer and participants arising from differences in educational background, social positioning and familiarity with the research process, particularly with pregnant women with limited formal education. Reflexive awareness was maintained throughout data collection to minimize potential bias and ensure that participants’ voices were authentically represented. During the data analysis, reflexivity was further maintained through quality checking of data, weekly team discussions and joint coding exercises. Initial data coding was conducted by SK with review from the broader research team. Discrepancies were discussed and resolved through consensus in weekly meetings, recognizing the team’s different perspectives. The team’s diverse disciplinary backgrounds facilitated critical reflection on interpretation of themes. Several team members had prior research experience on ANC quality in Nepal [23], and drew on comparisons between these studies during the weekly team meetings to guide areas of enquiry in the data collection and to deepen interpretation in the analysis. The team was conscious of prior assumptions and looked for negative or discordant cases within this study to keep the analysis grounded in the data. Further, the stakeholder meeting, which included some research participants, provided an additional opportunity for reflection and validation of findings.

### Inclusivity in global research

Additional information regarding the ethical, cultural, and scientific considerations specific to inclusivity in global research is included in the Supporting Information (see S1 Checklist).

## Results

The study took place in six health facilities offering ANC services in three municipalities (Table 1). In total, eight interviews with pregnant women, seven interviews with ANC providers and three interviews with local policymakers were conducted. The study settings and number of study participants are presented in Table 2.

**Table 2:** Study settings and number of study participants.

Type of health facilities	Number of health facilities	Total pregnant women interviewed	Total ANC providers interviewed
Health Post^a^	4	4	4
Primary Health Care Center	1	3	1
Municipal Hospital	1	1	2
**Total**	**6**	**8**	**7**

^a^All but one of the health posts did not have a birthing unit.

All the included health facilities offered childbirth services except for one health post. All seven providers interviewed were female, engaged primarily in providing ANC services to pregnant women visiting the health facilities and had working experiences ranging from three years to twenty years as an Auxiliary Nurse Midwife (ANM). The eight pregnant women participating in study were between four to nine months pregnant and ranged in age from 19-36 years old. The educational backgrounds of pregnant women varied from primary level to bachelor’s degree, including two participants who could not read and write. The participants involved pregnant women who were pregnant for the first, second and third times during their lifetime. Among the women, three were pregnant for the first time, three were pregnant for the second time and two for the third time.

Three overarching themes were developed during the data analysis. The first theme reflected how ANC providers and local policymakers perceived the new ANC model mainly as an increase in the number of visits, essentially for documentation, instead of focusing on the content of each visit. The second theme centered on how inadequate orientation to the new ANC model hindered the practices of providers on providing quality ANC care. The third theme centered around the barriers pregnant women experienced while attending multiple ANC visits. We discuss each theme in turn.

### Theme 1: ANC model as change in numbers, not content

This theme consistently emerged from interviews of policymakers and providers to reflect the common perception among ANC providers and policymakers who equated the new ANC model as solely about increased numbers of ANC visits rather than enhancing content and quality of care provided during visits.

Most of the ANC providers perceived the new ANC model as a mandate to document the number of ANC visits in the Safe Motherhood register provided to them. ANC providers consistently emphasized the importance of recording ANC visits in the register to follow the schedule of ANC visits. The documentation of completed visits was a key element of ANC in our observation of care. This was explained as meeting reporting requirements related to government financial incentives tied to ANC attendance: if the pregnant women completed eight recorded ANC visits, they would qualify for a small payment for attending scheduled ANC visits [18].

*“There is our schedule of 8 visits, and we are calling according to that schedule because when they (Pregnant women) come, if they do not visit as per the protocol then they will not get that benefit (ANC incentive) given by the government. Like, if their visit is as per the protocol then they would get 800 rupees after delivery as nutrition cost and we remind them to come as per the schedule, if they came as per schedule, they would get that benefit”. -ANC Provider 1*

While other purposes for the new ANC schedule of visits were acknowledged, eligibility for financial benefit was understood as central to adhering to the increased number of ANC visits.

*“If they (pregnant women) come as per the protocol, then we could find out their complications which might be there, and they would also get to have iron and calcium in regular basis and would not miss it and also the target of government would be achieved, and they would get the benefit(ANC incentive) given by government if they did not miss any one of the visits.” -ANC Provider 4*

Though all the ANC providers were aware that the increased visit could lead to screening of complications, we found that most of ANC providers were not aware of the reasons behind the model’s increased visits, and few ANC visits in our observations included recommended investigations for potential complications, for example urine testing at every visit. We found that most providers did not note any significant changes in content of the two different ANC models.

*“The whole checkup is the same, I did not see anything new on 8 ANCs like this should be done specially in this month. There might be and we might not have been trained on it, that is in its own place. We used to take weight before and measure it now as well, anomaly was done earlier as well and is done now too, now the VDRL (Venereal Disease Research Laboratory) lab tests were done earlier as well and now too. There is nothing like, this has been added specially in 8 visits.” -ANC Provider 7*

All interviewed municipal policymakers, whose role included supervising and monitoring implementation of the new ANC model, were also largely unfamiliar with the recommendations in the new ANC model guidelines beyond a change in number of visits.

*“No, there are no such differences, the tests which are done during the 4 visits, the same tests and counseling are done in 8 visits. The times of visit increased and in cases of weight increment they get to see the same activities 4 times more.”- Local Policymaker 2*

Similarly, one of the policymakers raised a question: “Is it necessary to train for all things?” upon inquiring about if there is provision of any ANC training packages to providers. This contrasted with concerns expressed by providers who described inadequate training on content of care as a major barrier to delivering quality care.

### Theme 2: Positive attitudes but limited orientation and resources

This theme captures the views expressed by providers and policymakers where both groups consistently expressed positive attitudes towards the new ANC model but identified gaps in governance to support its implementation. Stakeholders recognized the value of the increased visits along with potential to strengthen the quality of care. However, they simultaneously revealed limited orientation and inadequate dissemination of materials on content of care that could enhance communication about, monitoring of, and guideline implementation.

Consensus emerged across interviews as most providers in the health facilities stated that the increased number of ANC visits had improved their relationship with pregnant women, indicating that they believed they were providing good care. They also spoke of the potential benefits of more visits, reflecting the positive implications of the change in policy.

“*What I felt is, when they needed to come many times then there would be a little more intimacy (connection) and our service seeker would express their problems more openly and I felt that belongingness more. The more times they need to come, they would feel like this is their own institution, so, they need to visit it and tell us about problems, there is like... they tell their problems more eagerly. I feel like this might have happened and there is an increase in intimacy with them. I feel like it has been a little easier for me. -ANC Provider 5*

However, the tension between participant accounts of quality care and observational findings was evident, as we observed that the guidelines for the content of these visits were not followed adequately, such as history taking, examinations, investigations and health promotion activities as recommended. Most providers implementing the new ANC model were unfamiliar with the recommended content of care in the guideline but were aware of the shift in schedule.

*“The training given at first was 4 times and on 8 times checkup, what should be examined and why it became 8 times for this once orientation was given to nursing staff in the municipality. The other sister went at that time; she came and told us the things said there but let’s say the training was not given more carefully saying about what should be examined on 8 times to all nursing staff providing ANC in the institution.”- ANC Provider1*

Additionally, none of the providers were aware of the availability of facilitating materials, indicating all recommended services for the recommended eight ANC visits, highlighting significant gaps in knowledge and adherence to best ANC practice.

*“We have not known. It’s just said that 8 visits need to be made. Now we are calling regularly according to the time (schedule) and doing the things that we have been doing earlier but there has not been any specialized orientation to us on what should be done on which visit, nothing has happened to us.”- ANC Provider 3*

All providers also spoke of the need for more training/orientation on policy changes and stated the need for materials that would guide their practices while providing services.

*“We had talked about it (availability of information materials) as well, if there is availability of flip chart indicating what should be done on what time, sometime there will be much patient flow and we do not get chance to work freely, sometimes we may have confusion so if we had a book or a flipchart then I feel like it would be easier. No, we have not been provided with that.” – ANC provider 4*

Observations in health facilities found few facilitating materials (such as posters, flipcharts) on the content of new ANC model. Notably, one of the ANC providers, on her own initiative, created materials outlining the recommended content for ANC visits and was consistently observed providing ANC services in alignment. The provider claimed a lack of proper orientation from higher authorities; however, she described efforts to provide care according to recommended protocols and was observed to demonstrate an intrinsic motivation to enhance the quality of care.

*“It was said that the checkup should be done 8 times, the weeks were told briefly but it was not said in detail… We don’t have a protocol; we took it out on our own saying that it’s necessary. We also need to copy from others, I used to go to family planning (implying one of the other organization) and used to look there and I came back by capturing a photo and said them that it should be kept like this, after I said so, as there are junior sisters here who have more knowledge than me, I told them and they took it out accordingly and we pasted it.”- ANC Provider 6*

Amidst concerns about inadequate orientation impacting providers’ awareness and ability to deliver quality service, one local policymaker emphasized that the orientation/training before implementation of new ANC model lacked resource materials. However, the study team had located an implementation guide called “ANC to PNC Continuum of Care Guideline” online that was not available in any of the facilities during the time of observation. This was discussed in the stakeholders’ meeting where local policymakers as well as ANC providers were largely unaware of the document but responded positively to the resource material. The policymakers expressed a desire to use the materials as soon as possible and advocated for its proper dissemination to frontline providers to address communication gaps and support better supervision and monitoring of guideline implementation.

“*One of the reasons for arising this sort of gap(inconsistency in services of ANC) is, all of us became irresponsible, all of us mean those who developed the guideline, it should have been reached out to concerned places and assured whether that(guideline) reached there or not, for example, did they made it reach to my district or not and did I give it to local level or not? and even if you (Researcher) find it out after searching then the person providing the ANC check would have also found it if searched, so they (Providers) are also irresponsible. Meaning that all of us became irresponsible. – Local policymaker 3*

### Theme 3: Women’s effort undermined by limited service quality

This theme emerged consistently across interviews with both providers and pregnant women around the efforts made by pregnant women to seek ANC services despite challenges they faced in making those visits. While both groups valued the benefits of ANC visits, we observed that attending more visits did not necessarily translate into receiving quality care.

Most pregnant women were largely unfamiliar with the new ANC model and most of them explained that they had been visiting the health facilities for ANC appointments as per the instruction of providers. Some women noticed and appreciated an increase in the number of visits compared to previous pregnancy experiences. A pregnant woman who was seeking ANC in last month of the pregnancy compared the new model to her previous experience:

*“Now, I feel good. When I gave birth to my daughter, it was about 8,9 years ago. Now it’s said that one needs to check up to 8,9 months and till before having the baby. When given birth to my daughter I just came 2-3 times, video Xray and nothing else was done. Now it’s said that what things need to be done. I am doing whatever they say.” -Pregnant Woman 3*

Several pregnant women highlighted the challenges of attending ANC visits. They described the distance of the health facility from their place of residence, difficulty with access to transportation, difficulty to manage household chores, financial constraints when referred to facility that costs money, long waiting periods in the referred sites (tertiary level hospitals) and physiological challenges, such as pain in leg and back as major concerns for them adhering to multiple ANC visits.

*“Now I don’t have any person in my house to look after me, I have one daughter and I have been raising a few goats at home and I feel a little like, how many times to walk…like that. I have been called 4 times during my daughter’s turn and now it’s not like before also.”- Pregnant Woman 3*

Additionally, one of the ANC providers from health facility belonging to rural areas narrated the challenges of pregnant women on behalf of her experiences with ANC clients as:

*“How I feel is, Bichara (Helpless people) have many challenges because they need to come health facility by walking from far distances which is much difficult at later months of pregnancy. There is no vehicle facility for them, and they must walk uphill and downhill while carrying heavy pregnancy. So, when we ask them to revisit after two weeks, they say it is difficult for them to come so often. We send them by counselling about protocol and risks that might arise during the time. They feel bored and irritated.” -ANC Provider 6*

Although most of the pregnant women believed that ANC visits were beneficial for them, our observations revealed that not all facilities offered satisfactory ANC services. Observations of ANC found that the pregnant women attending the ANC visits were rarely provided with all content of care as mentioned in the national guideline (ANC to PNC Continuum of Care Guideline). Routine examinations, such as urine testing on each visit, and counselling were missing from most ANC consultations observed. One of the facilities was observed to offer minimal consultation time, as brief as three minutes of interaction between the provider and women.

Inconsistency in care across the health facilities was observed, but some of the ANC providers were offering personalized attention to the pregnant women. We observed that when some pregnant women reported any health issues, these providers extended consultation time and offered additional information and counselling tailored to the women’s reported health concern. However, in the absence of expressed concerns, counselling remained limited. The observed suboptimal content of ANC services highlighted that the efforts made by pregnant women to attend health facilities for ANC visits did not correspond to the quality of care provided.

## Discussion

The study explored the perspectives of policymakers, providers and pregnant women on the roll out of the new ANC model in Kavre district, Nepal. The findings suggested a critical gap between the understanding of model’s changes in both the purpose and frequency of ANC visits among all stakeholders, along with gaps in implementation of recommended content of care. These gaps could potentially contribute to suboptimal ANC quality despite considerable efforts made by pregnant women to complete additional ANC visits.

Our findings suggested that the providers and local policymakers had limited understanding of rationale behind change in the ANC model, seeing the model as solely about an increase in number of ANC visits. The WHO recommended ANC visit schedule emphasized frequent monitoring particularly on the final weeks of pregnancy to address risks like hypertension and other complications which emerge closer to the childbirth [24], but the recommended visit schedule from Government of Nepal deviated by including higher number of contacts earlier in the pregnancy, particularly in second trimester (see S3 Text) [2,5]. This schedule means that pregnant women will likely receive less frequent assessments in the third trimester compared to WHO recommendations. This discrepancy suggests that Nepal’s guideline might have missed the critical objective of frequent monitoring in final weeks of pregnancy, raising the important concern about whether this ANC schedule effectively addresses the key health issues as designed by WHO.

We found that providers and stakeholders had limited understanding about the content of care recommendations for each visit. This echoes findings from other low- and middle-income countries which showed that coverage of care components during ANC visits was often lower than high coverage of ANC attendance, suggesting that quality of care has not been given as much attention as achieving minimum numbers of visits [25–27]. A study in Ethiopia found similar challenges faced by providers and local policymakers during roll out of new ANC model, highlighting barriers such as: unavailability of guideline, inadequate pre-service clinical supervision, poor quality of in-service training, lack of regular mentorship and supervision and lack of monitoring and evaluation as on adhering to ANC guidelines despite the willingness of provider to provide care [10]. Similar to our findings, the study in Ethiopia emphasized that implementation of the new model requires comprehensive roll-out strategies to support adoption of the full guidance for content of care in the new ANC model [10]. Consequently, implementation with the corresponding orientation for providers and support for monitoring, not just the visit schedule but also the delivery of recommended ANC interventions, is needed to realize the intended benefits of the new ANC model.

Our findings align with Downe and colleagues’ 2016 review which highlighted that women pursue ANC with goals for a positive pregnancy and motherhood experience and this includes ensuring a healthy pregnancy, maintaining physical and sociocultural normality and receiving emotional and psychosocial support [3]. In our study, women were highly motivated to attend prescribed ANC visits, driven by strong desire to protect their health and that of their baby. They went to great effort to attend ANC visits more frequently under the new ANC model. Our findings emphasize the need to increase accessibility of ANC services but also responsive, respectful and person-centered ANC services in facilities which acknowledge women’s efforts in seeking services particularly in rural settings where geographical and transportation barriers further limit access. A study in Malawi found that the new ANC model was acceptable among women but noted the burden on women associated with increased ANC visits. Specifically, long distances to healthcare facilities and lack of transportation were major obstacles discussed by women [14], echoing findings from our study.

Our findings can be understood through key concepts of implementation science [28]. The observed shift towards increased number of ANC visits, without corresponding change in the content of care reflects challenges related to implementation fidelity [29]. The numerical components of the new ANC model were largely adopted while the functional core elements of investigations, physical examinations, health promotional activities and person-centered care were implemented less consistently, reflecting partial fidelity to the new ANC model. Our findings also highlighted the challenges in policy translation where WHO recommendations are adopted, however not fully operationalized within national guidelines and local implementation. We found the barriers in ‘coherence’, the sense-making work that people do to operationalize new practices as described in Normalization Process Theory, where there was an insufficient shared understanding among stakeholders about what was different in the new ANC model and its intended benefits [17]. How the new ANC model was understood shaped what individuals did to enact the changes in both the visits and the contents that were part of the ANC model. The limited shared coherence contributed to interpreting changes in policy as only an increase in visits rather than a shift towards improved quality and experience of care, evidenced by an observed focus of frontline providers in our study on reaching the recommended number of visits without substantive changes in the content of care provided.

Further, limited coherence at the policymaker’s level resulted in minimal dissemination efforts and a lack of supervisory and monitoring systems that could support strong implementation of the content elements of the new ANC model, in addition to the recommended visit schedule. This can lead to a coverage and quality gap, where expansion in service utilization does not necessarily translate into meaningful improvements in service delivery [27].

Future studies should examine implementation fidelity of the new ANC model at multiple sites to understand variation in rollout of the model, while recognizing that fidelity to the model reflects both changes in the content of care alongside the revised visit schedule. Nationally representative surveys, such as the NDHS, can be used to track coverage of numbers of ANC visits, but other sources of data are needed to capture fidelity to the visit schedule and content of care during each visit, which is intended to adapt to women’s needs and risks over the course of the pregnancy [2]. Quantitative assessments, such as those integrating multiple data sources including cross sectional facility-based surveys and patient exit interviews, could better capture content coverage of ANC visits to understand quality, completeness and responsiveness of services delivered [30].

### Strengths and limitations of the study

This study provides context-specific insights into the implementation of the new ANC model in selected municipalities of Nepal. A key strength of this study lies in the use of two qualitative methods of data collection, interviews and observations, to provide richer understanding of the context, and triangulation of findings, alongside the use of respondent validation in the stakeholder meeting to refine interpretations. The study also integrates multiple viewpoints, from pregnant women, ANC providers and local policymakers to explore implementation of the new ANC model.

Despite the strengths, the study has several limitations. This research emerged within a broader study which examined hygiene preparedness and urine testing practices in pregnancy in ANC settings. The data collection tools, and selection of participants were not originally designed to capture all dimensions of policy implementation, which shaped the depth and scope of this study. This was also a small-scale, exploratory study conducted in a single district and is not intended to represent national implementation patterns. There may be further dynamics of implementation of the new ANC model that were not captured in our study or issues that may be relevant in other districts or health system contexts, such as how WHO recommendations were adopted and understood within national-level guidelines. Additionally, as health facilities were purposively selected and included facilities with higher ANC caseloads, the findings may have limited transferability to lower volume facilities, which may differ in resources and service delivery. Further, the responses and practices of providers may have been shaped by social desirability bias in the interviews and the Hawthorne effect in the observations. Yet our observations noted inadequacies in quality of care even when under observation, suggesting that this was not a major issue in our study. Nevertheless, the findings provide context-specific insights that may be relevant to settings with similar health system characteristics implementing the new ANC model.

### Recommendations

Based on our findings, some actions are recommended to better implement the new ANC model. These recommendations were discussed at the stakeholders meeting conducted for the study, and all the stakeholders shared their commitment to ensure quality ANC services. The concerned authorities such as Ministry of Health and Population, NHTC, Family Welfare Division, Provincial Health Office and Municipal Health Offices should address communication gaps and disseminate detailed implementation guidelines in each facility and ensure that ANC providers are oriented to and understand what is new within the aims and content of the new ANC model. This should be supported by greater supervision and local monitoring of the ANC model implementation that goes beyond documentation of numbers of visits to ensure delivery of recommended content of care. The coordinated actions across system levels can help address gaps in the quality antenatal care for improved maternal and perinatal outcomes. Further, these recommendations offer lessons for future maternal health policy reform in Nepal and comparable contexts, in the importance of dissemination and orientation to develop shared understanding of the policy’s objectives and appropriate monitoring of its implementation.

## Conclusions

The study integrated findings from facility observations and interviews with stakeholders in Kavre district, Nepal on the implementation of the new ANC model. Implementation of the new ANC model primarily focused on increasing the number of visits, where limited knowledge of providers on the recommended content of each visit led to the observed suboptimal quality of ANC visits. It is crucial for providers to make each ANC visit meaningful, especially given the access barriers pregnant women face. Improved training and orientation for providers could support greater awareness of the purpose of the new ANC model and greater adherence to guideline-recommended care. Offering tangible tools such as facilitation materials, flip charts, posters, pamphlets are essential to assist providers in adhering to ANC protocols. However, these efforts must be complemented by supportive monitoring, supervision and a conducive environment. Addressing gaps in content of care like those observed in our study is essential to realize the intended benefits of the new ANC model to improve maternal and perinatal outcomes and ensure a positive pregnancy experience. These findings reflect the early implementation experiences within a single district and may evolve over time with strengthened support. Nevertheless, the insights provide valuable indications of potential challenges in translating national guidelines into frontline service delivery.

## Supporting information

S1 Text Topic guides. This appendix contains the detailed topic guides used for interviewing pregnant women, ANC providers and local policymakers.
(DOCX)

S2 Text Observation checklist. This appendix includes the semi-structured checklist used during facility observations.
(DOCX)

S3 Text Comparison of WHO and Nepal ANC Timing Recommendations. This appendix presents a side-by-side comparison of WHO-recommended timing of ANC contacts and the national ANC schedule adopted in Nepal.
(DOCX)

S1 Checklist Inclusivity in global research. (DOCX)

S2 Checklist Consolidated criteria for reporting qualitative studies (COREQ) checklist. (DOCX)
