# Supplementary material for: From visit counts to quality care: A qualitative study on early implementation of a new model for antenatal care in Kavre district, Nepal
Source: PLOS Glob Public Health. 2026 Oct 5;6(10):e0005076. doi: 10.1371/journal.pgph.0005076 (PMC13637887; doi:10.1371/journal.pgph.0005076)
Supplement: Supplementary file 1 [file pgph.0005076.s001.docx]

**Topic guide for Pregnant Women**

**Section 1: Introduction (Rapport building and demographics)**

- Introduce yourself (interviewer)
- Can you tell me about yourself? *(probes- Name, age, educational background, address, duration of pregnancy)*
- Is this your first pregnancy?

**Section 2: Understanding Hygiene and Maternity Care of specific health facility**

- For how long have you been visiting this health institution to seek antenatal care? Have you visited any other places for antenatal care?
  - Where did you register for antenatal care?
  - Probe: Have you been referred to any other places for further investigations or services? If so, what has been your experience attending for antenatal care at other facilities?
- How would you describe the quality of care and the environment at this facility? (probe: have you had a comfortable place to wait, privacy during consultation, easy to get all tests and medicines needed, etc)
- Are there any specific aspects of the facility’s environment or services that you believe could be improved?

**Section 3: Services received (Focus on Urine Screening)**

- What are the services you received today?
- What is important to you when you come for an antenatal care check? (Probe: how satisfied are you with your visit today?)
- Women are often asked to give a urine sample for different tests when they are pregnant. *Were you asked to give a urine sample during today’s visit? (If not today, then in previous visits?*
- *How did you feel about that process? (probe: location of toilet, condition of toilet, comfortness of toilet, availability of soap and water for handwashing after collecting urine sample)*
- *How would you describe the cleanliness and hygiene infrastructure of this healthcare facility? Has it met your expectations?*
- *Have you faced any challenges or concerns related to hygiene and cleanliness during your ANC visit?*
- *Were you told what the urine sample was for?*
- *Was it comfortable for you to provide a urine sample? (If yes why, if not why?)*
- Have the ANMs or doctors talked with you about how many times you should visit during pregnancy for ANC check ups? (if yes, have you heard about 8 ANC visits, what do you think about the government recommendation to attend 8 antenatal check-ups)
- Have you made plans for where you will give birth? Where and why have you chosen that location?

**Section 4: Closing remarks**

- Are there any additional services that you believe should be available for pregnant women at this health care facility?
- Is there anything else that you would like to share about your experiences with antenatal care in regard to ANC?

**Thank your participant for their valuable time and ask if they have any queries.**

**Topic guide for ANC Providers**

**Section 1: Introduction (Rapport building and demographics)**

1. Can you please give me your short introduction? (Name,age,designation, working experience, roles and responsibility within health facility)
2. What is your involvement in antenatal care?

**Section 2: Facility design and hygiene preparedness**

1. How would you describe the overall quality of facilities here?
2. Do you think your facility/ANC setting is well organized for pregnant women of all kinds? (Probe for yes/No)
   a. During busy periods, how do you manage patient flow?
3. Are all the facilities necessary for the maternal service available in this health facility?
4. When was the last time infrastructure improvements were made to this facility (and what were they)?
5. What are the improvements made specifically in terms of ANC settings?
6. How would you describe the overall state of water, sanitation and hygiene in your health facility? *(Probe: Toilet, Basin, availability of soap, sanitizer….)*
   a. Are the hand washing and toilet facilities convenient to use?
7. What are the current efforts that you are making to ensure hygiene preparedness of ANC settings of the health facility?
8. What do you think are the key challenges or barriers that your health facility is facing in regard to hygiene preparedness, particularly in ANC ? (probe for financial, social, logistical, technological, staffing and other forms of barriers) (example: are toilets pregnant women friendly?Is there an availability of washing basin, soap, water?Is it possible for you to wash your hands in between procedures?)

**Section 4: Antenatal Urine Screenin**g

1. Can you tell me about the ANC visits that you recommend to your clients?
2. What are the investigations prescribed in each of the visits? Are all the investigations available here?
3. Can you please tell me more about what urine screening services are offered to pregnant women? (What tests are offered? At what point in the pregnancy?)
4. When asking pregnant women to give a urine sample for screening, what is the process?
5. How important is urine screening for pregnant women?
6. What challenges do you face in offering urine screening service to women during ANC?
7. As GoN has recommended 8 ANC visits? What is the current status of these recommended visits in this clinic?
8. What do you think are the implications of these 8 scheduled ANC visits?

**Section 5: Closing Remarks**

1. Are there any suggestions you would like to recommend in regard to improving the facility?
2. Are there anything else you would like to add?

**Thanks to the participant for their time**

**Topic guide for Policymakers**

**Section 1: Introduction (Rapport building and demographics)**

1. Can you please give your short introduction? *(Name, age, designation, working experience, roles and responsibilities)*
2. For how long have you been working for the municipality?
3. What is your role?

**Section 2: Understanding the current perception of hygiene infrastructure in health facilities within municipality**

1. How would you describe the overall state of water, sanitation and hygiene in the healthcare facilities of your municipality/ district/ Province? *(Probe: Toilet, Basin, availability of water, soap, sanitizer,….)*
2. Can you please tell me about any monitoring or other activities in place to check water, sanitation and hygiene infrastructure in health facilities?
3. What standards are used to assess whether water, sanitation and hygiene facilities are sufficient?

**Section 3: Process of upgrading facilities**

1. We understand there is a government commitment to have a hospital in all of Nepal’s municipalities. Can you tell us about that process?
2. Do you have any health facilities that are in the process of upgrading?
3. How are facilities selected for upgrading or infrastructure improvements? (Probe for political and logistical process)
4. Who is involved in making those decisions?
5. What data or evidence is used in making investments to improve facilities?
6. Who is involved in designing new facility infrastructure?
7. How is facility layout or design considered during improvements to health facility infrastructure?
8. How are water, sanitation and hygiene issues considered when upgrading health facilities?

**Section 3: Challenges/ Barriers/ Facilitators**

1. What challenges does your municipality face in terms of health facility infrastructure and services?
2. What do you think are the key challenges or barriers that your municipality is facing in regard to addressing those challenges? (probe for financial, social, logistical, technological and other forms of barriers)

**Section 4: Closing remarks**

1. Are there any suggestions you have for improving health infrastructure or services in your municipality?
2. Is there anything else you would like to add?

**Thanks to the participant for the time**
