# Supplementary material for: From visit counts to quality care: A qualitative study on early implementation of a new model for antenatal care in Kavre district, Nepal
Source: PLOS Glob Public Health. 2026 Oct 5;6(10):e0005076. doi: 10.1371/journal.pgph.0005076 (PMC13637887; doi:10.1371/journal.pgph.0005076)
Supplement: Supplementary file 2 [file pgph.0005076.s002.docx]

**Work Guide for Facility Observation**

**Study Aim:** To explore whether and how the facility’s physical environment constrains or enables the provision of maternity services, including urine screening, and hand hygiene practices.

**Approach**:

Note down observations throughout the day, organizing them under the topic areas provided. **Take photos to document** **aspects** of the facility layout or infrastructure you find interesting and relevant to WASH and maternity services, taking care to **avoid including people – and particularly individuals’ faces – in any photographs.**

**Note: Write *Reported if things are noted down by asking and not observing**

**Topic Area 1: WASH Infrastructure**

| S.N | Description | Observation | Remarks (To note down any extra data) | Hint |
| --- | --- | --- | --- | --- |
|  |  |  |  |  |
| 1 | Note whether and **who is a staff focal** person for WASH/Infection prevention and control (IPC) practices at the facility. |  |  | Name of staff |
| 2 | **What is the** **main water supply** for the facility? and where is it located? |  |  | Piped into facility  Public Tap  Tube Well/borehole  Dug well (protected/not)  Rainwater  Bottled water  Tanker Truck  Surface water ( River, streams, reservoirs) |
| 3 | **Where** is the main water supply located? |  |  | On-premises  Within 500m of facility  Beyond 500 m of facility |
| 4 | Is water available at the time of the visit? |  |  | Yes/No (Check for running water in the tap) |
| 5 | What **type of toilets/latrines** are at the facility for patients? |  |  | (Flush toilet/Squat toilet.)  Take photo |
| 6 | Are there toilets **that are in single-sex or gender-neutral rooms?** |  |  | Common toilet for all sex/ separated/  Take photo |
| 7 | Is at least one toilet usable? |  |  | (Available/Functional/Private )  Available: have access/doors unlocked/key accessible  Functional: Water available/no cracks or leaks/no pit blocked  Private: locked doors |
| 8 | Are there **toilets that are dedicated to staff?** |  |  | Yes/No |
| 9 | Are there **toilets in the area where maternal and newborn services** are provided? |  |  | Yes/No |
| 10 | Is there **at least one toilet** in the area where maternal and newborn services are provided? |  |  | Yes/No (Note down the number of toilets) **If no jump to 11** |
| 10.1 | Is that toilet **usable at the time of the survey?** |  |  | Yes/No (functional and private) |
| 10.2 | **Accessible to women in labor** or advanced stages of pregnancy (no steps, handrails and space for assistance) |  |  | Note down (Take a photo)  Can be accessed without stairs  has handrails/space for assistance  Should be within service provided area |
| 10.3 | **Is that toilet single-sex?** |  |  | Yes/No (If no, what?) |
| 11 | **Is there a functional hand-washing** facility at all toilets on the day of the visit? |  |  | Yes/No  Functional=availability of water and soap(bar/liquid) and also note if there is alcohol based handrub |
| 12 | Is there a **functional hand-washing facility inside the ANC examination room** on the day of the visit? |  |  | Yes/No |

**If the facility offers delivery services,** additionally observe the following (In the delivery room)

| S. N | Description | Observation | Remarks | Hint |
| --- | --- | --- | --- | --- |
| 1 | Types of water supply in the delivery room |  |  | Note down |
| 2 | Water in the delivery room is currently available |  |  | Yes/No |
| 3 | Toilet available to women during and after labor |  |  | Yes/No (**If no jump to 6)** |
| 3.1 | If yes, is it? |  |  | (Single sex/gender neutral) |
| 4 | Is the toilet usable (available, functional, private) |  |  |  |
| 5 | Is the toilet accessible for women in labour ? |  |  | Yes/No  (Take a photo) |
| 6 | Functional hand washing facility in the delivery room |  |  | Yes  Partially (lacking soap or water)  No  (Take photo) |
| 7 | Sterile materials available in the delivery room | Sterile blade  Sterile cord tie  Clean surface to deliver  Disposable gloves |  | (Tick all that apply) |
| 8 | Functional shower or bathing area available where maternal and newborn services are provided: |  |  | Yes/No  If there is piped water in the bathing area, check that taps are working. If there is no piped water check that containers are available (or delivered to the bathing area when needed). |
| 9 | Sharps, infectious and general waste are safely separated into three bins in the delivery room: |  |  | Yes  Somewhat  No  The bins should be colour-coded and/or clearly labelled, no more than three  quarters (75%) full, and each bin should not contain waste other than that  corresponding to its label.  (Take a photo) |
| 10 | Protocols for cleaning the delivery room |  |  | (Delivery bed, floor, sink, blood/bodily fluids spillage)  (Take a photo) |
| 11 | Cleaning schedules are available: |  |  | Yes  Partially (not all protocols or no schedule)  No  (Take a photo) |

To note down anything that seems linked with our area of interest:

**Topic area 2: Facility design and functioning**

1. **Draw an approximate map or floorplan of the facility and mark the location of key items of interest**

- including ANC examination room(s)
- patient waiting areas
- patient and/or staff toilets
- hand washing stations (sinks or hand gel dispensers)
- laboratory facilities
- supply cupboards, etc

Space to draw and locate things:

2. Observe how the spaces link together, where crowding occurs, and where people spend the most time. How is space organized/divided? (Sketch it out)
3. Observe selected individuals (i.e., the ANC provider on duty) and discretely follow their movements continuously throughout the facility and map these movements to the floor plan.
4. Observe where and when healthcare providers wash their hands. (Note it down)

**Topic Area 3: Antenatal care (ANC) services and urine screening**

1. Map how women move through the facility to receive an ANC visit: (note it down or sketch it out)

Things to observe: what is done, where, and by whom. i.e., where does she wait, where are the different tests done and by whom, where is the physical exam done and by whom?

| Questions | Observation | Remarks (note out any reflections) |
| --- | --- | --- |
| Where does she wait? |  |  |
| What are the different tests done? |  |  |
| Tests done by whom? |  |  |
| Where is the physical examination done? |  |  |
| Physical examination done by whom? |  |  |

2. Observe how ANC visits are organized: for example, is the woman sent to the lab (or toilet to collect a urine sample) at any point during the visit?

| Questions | Observation | Remarks |
| --- | --- | --- |
| Is the woman sent to a lab? (Urine collection) |  |  |
| Is the woman sent to a toilet? (Urine collection) |  |  |
| When does this happen? (Before examination, part way through or anything you observe) |  |  |
| Patterns noticed |  |  |
|  |  |  |

4. Note where key equipment and infrastructure for antenatal care are stored and accessed. What objects are interacted with most and least and by whom and when?

Where?.............................................................................................................................................................

What objects?...............................................................................................................................................

Objects interacted by whom?..................................................................................................................................

When were the objects interacted with?.............................................................................................................................................

5. Note whether staff wash their hands between examinations with different ANC patients.

Yes/No………………………………………………………

If yes, how many times?.............................................................

Just by water or soap as well?...............................................................

6. **Note down things that seem to be in our area of interest: (reflection)**
