# Supplementary material for: From visit counts to quality care: A qualitative study on early implementation of a new model for antenatal care in Kavre district, Nepal
Source: PLOS Glob Public Health. 2026 Oct 5;6(10):e0005076. doi: 10.1371/journal.pgph.0005076 (PMC13637887; doi:10.1371/journal.pgph.0005076)
Supplement: Supplementary file 3 [file pgph.0005076.s003.docx]

**Comparing the 8 recommended timing of ANC visits in WHO and Nepal guidelines**

| **Timing of visits** | **WHO 8+ Recommended schedule** [1] | **Nepal new ANC schedule** [2] |
| --- | --- | --- |
| Visit 1 | Up to 12 weeks | Up to 12 weeks |
| Visit 2 | 20 weeks | Up to 16 weeks |
| Visit 3 | 26 weeks | 20-24 weeks |
| Visit 4 | 30 weeks | 28 weeks |
| Visit 5 | 34 weeks | 32 weeks |
| Visit 6 | 36 weeks | 34 weeks |
| Visit 7 | 38 weeks | 36 weeks |
| Visit 8 | 40 weeks | 38-40 weeks |

**References:**

1.	Organization world health. WHO Recommendation on Antenatal care for positive pregnancy experience. WHO Recommendation on Antenatal care for positive pregnancy experience. 2016. Doi: ISBN 978 92 4 154991 2

2.	DOHS/Family Welfare Division. ANC to PNC Continuum of Care Guideline. 2080.
