# Supplementary material for: From visit counts to quality care: A qualitative study on early implementation of a new model for antenatal care in Kavre district, Nepal
Source: PLOS Glob Public Health. 2026 Oct 5;6(10):e0005076. doi: 10.1371/journal.pgph.0005076 (PMC13637887; doi:10.1371/journal.pgph.0005076)
Supplement: Supplementary file 5 [file pgph.0005076.s005.docx]

**Consolidated criteria for reporting qualitative studies (COREQ) checklist**

|  | **Item** | **Location in manuscript** |
| --- | --- | --- |
|  |  | Page X, Line No. X |
| **Domain 1: Research team and reflexivity** | | |
| 1 | Interviewer/facilitators | Page 7, Line No. 128  Page 9, Line No. 160-161 |
| 2 | Credentials | Page 9, Line No. 160-161 |
| 3 | Occupation | Page 9, Line No. 160-161 |
| 4 | Gender | Page 9, Line No. 160 |
| 5 | Experience and training | Page 9, Line No. 160-161  Page 11, Line No. 229-230,  Page 12, Line No. 240-241 |
| **Relationship with participants** | | |
| 6 | Relationship established | Page 7, Line No. 128-130 |
| 7 | Participant knowledge of the interviewer | Page 7, Line No. 128-130 |
| 8 | Interviewer characteristics | N/A |
| **Domain 2: Study Design** | | |
| **Theoretical Framework** | | |
| 9 | Methodological orientation and theory | Page 10/11, Line No. 204-216 |
| **Participant Selection** | | |
| 10 | Sampling | Page 8, Line No. 134-148, 150-151 |
| 11 | Method of approach | Page 9, Line No. 161-165 |
| 12 | Sample Size | Page 8, Line No. 137, 142, 147 |
| 13 | Non-Participation | N/A |
| **Setting** | | |
| 14 | Setting of data collection | Page 7, Line No. 130-131 |
| 15 | Presence of non-participants | N/A |
| 16 | Description of sample | Page 13, Line No. 257-266 |
| **Data collection** | | |
| 17 | Interview guide | Page 9, Line No. 179-182 |
| 18 | Repeat interviews | Page 8, Line No. 151-155 |
| 19 | Audio/visual recording | Page 10, Line No. 187-189 |
| 20 | Field notes | Page 6, Line No. 107-108 |
| 21 | Duration | Page 9, Line No. 178 |
| 22 | Data saturation | Page 8, Line No. 151-155 |
| 23 | Transcripts returned | N/A |
| **Analysis and findings** | | |
| 24 | Number of data coders | Page 10, Line No. 192-193 |
| 25 | Description of the coding tree | N/A |
| 26 | Derivation of themes | Page 13, Line No. 267 |
| 27 | Software | Page 10, Line No. 198-199 |
| 28 | Participant checking | Page 11, Line No. 222-227  Page 13, Line No. 248-250 |
| **Reporting** | | |
| 29 | Quotations presented | Page 10, Line No. 200-202 |
| 30 | Data and findings consistent | Page 14, Line No. 275-278  Page 16, Line No. 324-329  Page 19, Line No. 398-401 |
| 31 | Clarity of major themes | Page 14, Line No. 274  Page 16, Line No. 323  Page 19, Line No. 397 |
| 32 | Clarity of minor themes | N/A |
